# The Rho guanine nucleotide exchange factor ARHGEF5 promotes tumor malignancy via epithelial–mesenchymal transition

**DOI:** 10.1038/oncsis.2016.59

**Published:** 2016-09-12

**Authors:** Y Komiya, Y Onodera, M Kuroiwa, S Nomimura, Y Kubo, J-M Nam, K Kajiwara, S Nada, C Oneyama, H Sabe, M Okada

**Affiliations:** 1 Department of Oncogene Research and Research Institute for Microbial Diseases, Osaka University, Osaka, Japan; 2 Department of Molecular Biology, Hokkaido University Graduate School of Medicine, Hokkaido, Japan; 3 Department of Radiation Medicine, Hokkaido University Graduate School of Medicine, Hokkaido, Japan; 4 Global Station for Quantum Medical Science and Engineering, Global Institution for Collaborative Research and Education (GI-CoRE), Hokkaido University, Hokkaido, Japan; 5 Division of Microbiology and Oncology, Aichi Cancer Center Research Institute, Aichi, Japan

## Abstract

Epithelial tumor cells often acquire malignant properties, such as invasion/metastasis and uncontrolled cell growth, by undergoing epithelial–mesenchymal transition (EMT). However, the mechanisms by which EMT contributes to malignant progression remain elusive. Here we show that the Rho guanine nucleotide exchange factor (GEF) ARHGEF5 promotes tumor malignancy in a manner dependent on EMT status. We previously identified ARHGEF5, a member of the Dbl family of GEFs, as a multifunctional mediator of Src-induced cell invasion and tumor growth. In the present study, ARHGEF5 was upregulated during tumor growth factor-β-induced EMT in human epithelial MCF10A cells, and promoted cell migration by activating the Rho-ROCK pathway. ARHGEF5 was necessary for the invasive and *in vivo* metastatic activity of human colorectal cancer HCT116 cells. These findings underscore the crucial role of ARHGEF5 in cell migration and invasion/metastasis. An *in vivo* tumorigenesis assay revealed that ARHGEF5 had the potential to promote tumor growth via the phosphatidylinositol 3-kinase (PI3K) pathway. However, ARHGEF5 was not required for tumor growth in epithelial-like human colorectal cancer HCT116 and HT29 cells, whereas the growth of mesenchymal-like SW480 and SW620 cells depended on ARHGEF5. Induction of EMT by tumor necrosis factor-α or Slug in HCT116 cells resulted in the dependence of tumor growth on ARHGEF5. In these mesenchymal-like cells, Akt was activated via ARHGEF5 and its activity was required for tumor growth. Analysis of a transcriptome data set revealed that the combination of ARHGEF5 upregulation and E-cadherin downregulation or Snail upregulation was significantly correlated with poor prognosis in patients with colorectal cancers. Taken together, our findings suggest that EMT-induced ARHGEF5 activation contributes to the progression of tumor malignancy. ARHGEF5 may serve as a potential therapeutic target in a subset of malignant tumors that have undergone EMT.

## Introduction

The malignant progression of tumor cells is associated with acquisition of invasive and metastatic properties and uncontrolled cell growth.^1, 2^ Over the course of this process, epithelial tumor cells often undergo epithelial–mesenchymal transition (EMT),^3, 4, 5, 6^ a reversible phenotypic change that takes place during embryonic development, wound healing, and malignant progression. EMT is generally characterized by the downregulation of epithelial markers such as E-cadherin and occludin, and the upregulation of mesenchymal markers such as N-cadherin, vimentin and matrix metalloproteinase. During EMT, epithelial cells lose cell–cell junctions and apicobasal polarity, and acquire invasive phenotypes that are essential for metastatic spread. These directional shifts in gene expression are regulated by several transcription factors, including Snail, Slug and ZEB1/2; these are induced by cell signaling activated by cytokines and growth factors such as tumor growth factor-β (TGF-β),^7^ tumor necrosis factor-α (TNF-α),^8, 9^ epidermal growth factor^10^ and hepatocyte growth factor.^10^ Mutations and/or epigenetic alterations in these EMT driver genes have a role in EMT induction,^11, 12^ and they correlate with disease relapse and survival in patients with cancer. These observations indicate that an aberrant EMT process leads to poor clinical outcomes.^13, 14^ Furthermore, suppression of EMT can increase sensitivity to anticancer drugs.^15, 16^ Therefore, the identification of EMT characteristics and inhibitors of EMT-related molecules could potentially contribute to the treatment of cancer.

The invasive and metastatic potential of tumor cells is partly regulated by the Src family of non-receptor tyrosine kinases.^17^ Src is upregulated in various human cancers, resulting in the deregulated turnover of focal adhesions and cytoskeletal remodeling, thereby promoting cell adhesion and migration.^18, 19^ Src also contributes to tumor invasion by inducing the expression of matrix metalloproteinases via the signal transducer and activator of transcription 3 pathway.^20^ In a previous study, we dissected Src signaling using an inducible system for Src activation^21^ and found that the Rho guanine nucleotide exchange factor (GEF) ARHGEF5, a member of the Dbl family of Rho GEFs, is crucial for Src-induced formation of podosomes (or invadopodia).^21^ Podosomes are protruding membrane structures with the ability to degrade the extracellular matrix, and their formation is tightly associated with the invasive potential of tumor cells.^22, 23^ Furthermore, we showed that ARHGEF5 is phosphorylated by Src, resulting in the elevation of GEF activity toward RhoA.^21, 24^ These results suggest that ARHGEF5 mediates the Src oncogenic signal to promote invasive potential via the Rho pathway.^25^ ARHGEF5 is induced by Smad signals during TGF-β-induced mesenchymal transition of endothelial cells (EndMT),^26^ suggesting a role for ARHGEF5 in the TGF-β-induced cytoskeletal remodeling. Furthermore, ARHGEF5 was identified as an important factor in the chemotaxis of macrophage-related cells by small interfering RNA (siRNA) screening.^27^ Despite functional compensation by related GEFs, ARHGEF5-null mice exhibited an impaired chemotaxis of immature dendritic cells and reduced migration of dendritic cells from the skin to the lymph nodes.^27^ Taken together, these observations highlight the crucial role of ARHGEF5 in regulating cytoskeletal remodeling linked to cell migration and invasion.

The *ARHGEF5* gene was originally identified as an oncogene by focus formation assays in NIH3T3 cells.^28, 29^ Recent reports showed that ARHGEF5 upregulation promotes tumorigenesis,^30^ and that coexpression of ARHGEF5 and Src is associated with poor prognosis of patients with non-small-cell lung cancer.^31^ In addition, ARHGEF5 overexpression markedly increase Src-induced tumor growth,^21^ implying that the Src-ARHGEF5 pathway has important roles not only in invasion and metastasis but also in tumor growth. However, the function and regulation of this pathway during malignant progression remain elusive.

In the present study, we focused our analysis on the function of ARHGEF5 in the context of EMT because of its potential link to malignant progression. We show that ARHGEF5 is functionally upregulated during EMT and promotes invasion/metastasis and tumor growth, particularly in cells that have acquired mesenchymal phenotypes. In support of this, analysis of a transcriptome data set revealed that the combination of ARHGEF5 upregulation and E-cadherin downregulation or Snail upregulation is significantly correlated with poor prognosis in patients with colorectal cancers. These findings indicate that EMT-induced ARHGEF5 activation contributes to the progression of tumor malignancy. ARHGEF5 may serve as a potential therapeutic target of a subset of malignant tumors that have undergone EMT.

## Results

### ARHGEF5 and Src are upregulated during TGF-β-induced EMT in MCF10A cells

To determine the relevance of ARHGEF5 to EMT, the effects of TGF-β-induced EMT on the expression and function of ARHGEF5 were examined using the human breast epithelial MCF10A cell line as a model system. TGF-β treatment induced apparent morphological changes in MCF10A cells, which were accompanied by E-cadherin downregulation, N-cadherin upregulation, actin cytoskeleton rearrangement and the formation of podosome-like structures (Supplementary Figures S1A and B). In addition, TGF-β treatment strongly promoted cell migration (Supplementary Figure S1C). These observations confirmed that TGF-β induced EMT in these cells.

**Figure 1:**
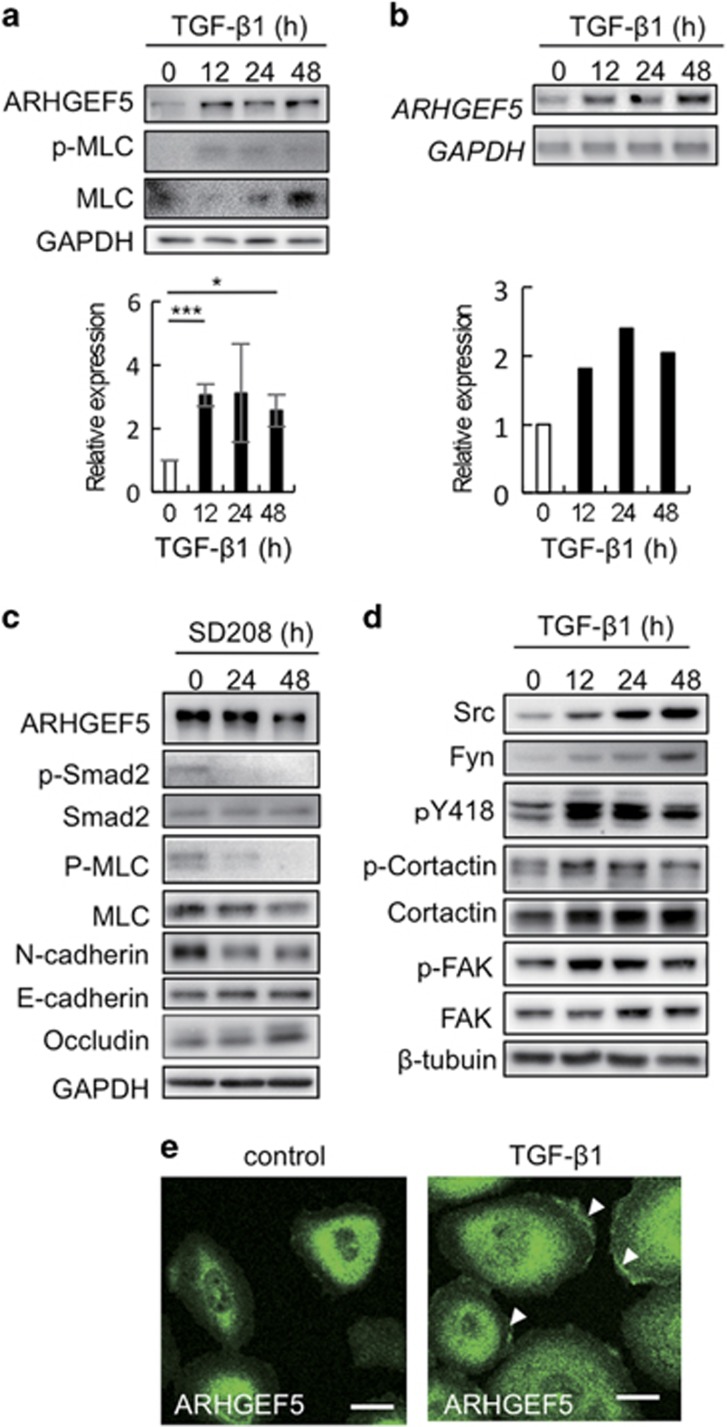
ARHGEF5 and Src are upregulated during TGF-β-induced EMT in MCF10A cells. (**a**) Expression of ARHGEF5 and phospho-MLC in TGF-β1-treated MCF10A cells was analyzed by western blotting. Values represent the mean±s.d. (*n*=3, *P<0.05, ***P<0.001). (**b**) Expression of *ARHGEF5* mRNA in TGF-β1-treated MCF10A cells was assessed by reverse transcription–polymerase chain reaction (RT–PCR). (**c**) MCF10A cells were treated with SD208 and the levels of the indicated proteins analyzed by western blotting. (**d**) Expression of the indicated proteins in TGF-β1-treated MCF10A cells was analyzed by western blotting. (**e**) MCF10A cells treated with or without TGF-β1 for 48 h were immunostained for ARHGEF5. White arrowheads indicate the ARHGEF5-positive areas in lamellipodia. Scale bar: 20 μm.

These processes were accompanied by ARHGEF5 upregulation at the protein and mRNA levels (Figures 1a and b). As TGF-β signaling is basally activated in MCF10A cells via the autocrine action of TGF-β,^32^ blockade of TGF-β signaling with the TGF-β receptor inhibitor SD208 downregulated ARHGEF5 expression (Figure 1c). The phosphorylation of myosin light chain (MLC) was altered in parallel with ARHGEF5 expression levels in response to treatment with TGF-β and SD208 (Figures 1a and c), indicating that ARHGEF5 is involved in the activation of the Rho-ROCK pathway.^25^

To examine the functional link between ARHGEF5 and Src, we investigated the expression and function of Src family kinases during TGF-β-induced EMT. The expression and activity (pY418 signals) of Src and Fyn, which function at focal adhesions,^17^ were elevated during EMT, concomitant with increased phosphorylation of their substrates, cortactin and FAK (focal adhesion kinase) (Figure 1d). Induction of EMT resulted in the accumulation of ARHGEF5 in regions near the edges of lamellipodia, where focal adhesion molecules, including tyrosine phosphorylated proteins and actin fibers, are directed (Figure 1e).^33^ These findings demonstrate that ARHGEF5 and Src are upregulated and accumulate at sites of cell adhesion during TGF-β-induced EMT.

### ARHGEF5 KD attenuates TGF-β-induced EMT phenotypes and cell migration in MCF10A cells

**Figure 2:**
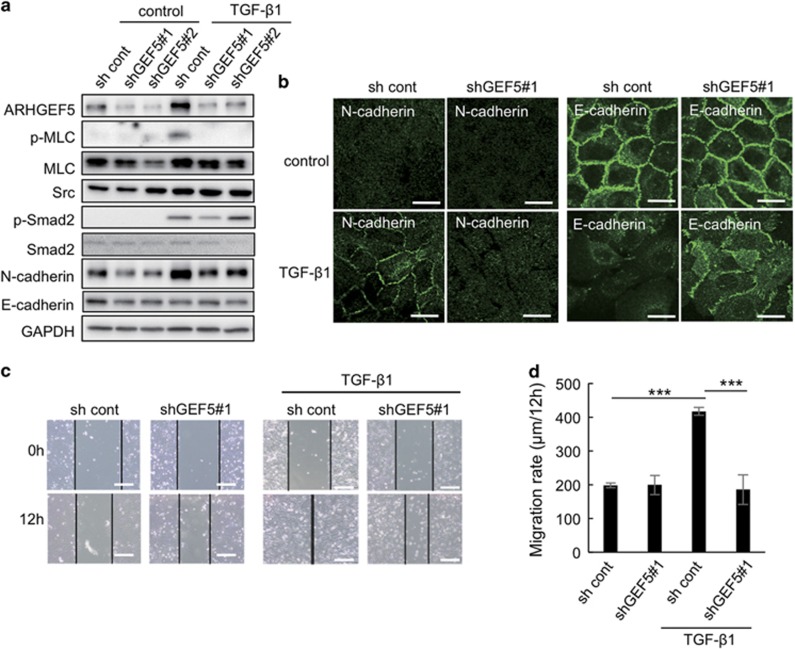
ARHGEF5 KD attenuates TGF-β-induced EMT and cell migration in MCF10A cells. (**a**) ARHGEF5 was knocked down with shRNAs (shGEF5 no. 1 and shGEF5 no. 2) in MCF10A cells. Mock and ARHGEF5 KD cells were treated with or without TGF-β1 for 24 h and the levels of the indicated proteins analyzed by western blotting. (**b**) Mock and ARHGEF5 KD cells were treated with or without TGF-β1 for 48 h and the cells stained with anti-N-cadherin or anti-E-cadherin. Scale bar: 20 μm. (**c**) Mock and ARHGEF5 KD cells were treated with or without TGF-β1 for 24 h and subjected to wound-healing assays. Scale bar: 200 μm. (**d**) The migration rates of the indicated cells are shown. Values represent the mean±s.d. (*n*=3, ***P<0.001).

To verify the contribution of ARHGEF5 to EMT, we examined the effects of ARHGEF5 knockdown (KD) on cell morphology and motility. ARHGEF5 KD suppressed EMT-induced MLC phosphorylation and MLC protein levels (Figure 2a). Immunofluorescence analyses revealed that ARHGEF5 KD attenuated N-cadherin membrane presentation and loss of E-cadherin-mediated cell–cell contacts (Figure 2b). Wound-healing assays revealed that ARHGEF5 KD suppressed cell migration in TGF-β-treated MCF10A cells, whereas it did not significantly affect cell migration in untreated cells (Figures 2c and d). Similar suppressive effects on TGF-β-induced EMT phenotypes were observed in response to ROCK inhibition by Y27632 (Supplementary Figures S2A–C). Furthermore, ARHGEF5 overexpression induced hyperphosphorylation of MLC, but was neutralized by treatment with Y27632 (Supplementary Figure S2D). These results suggest that ARHGEF5 is involved in the progression of EMT phenotypes via activation of the Rho-ROCK pathway. However, ARHGEF5 KD did not affect the TGF-β-mediated induction of EMT-related transcription factors, including SNAI1/2/3, TWIST1/2 and ZEB1/2 (Supplementary Figure S3), suggesting that ARHGEF5 functions downstream of these transcription factors in the TGF-β signaling pathway.

### ARHGEF5 promotes cell migration and invasion in colorectal cancer cells

**Figure 3:**
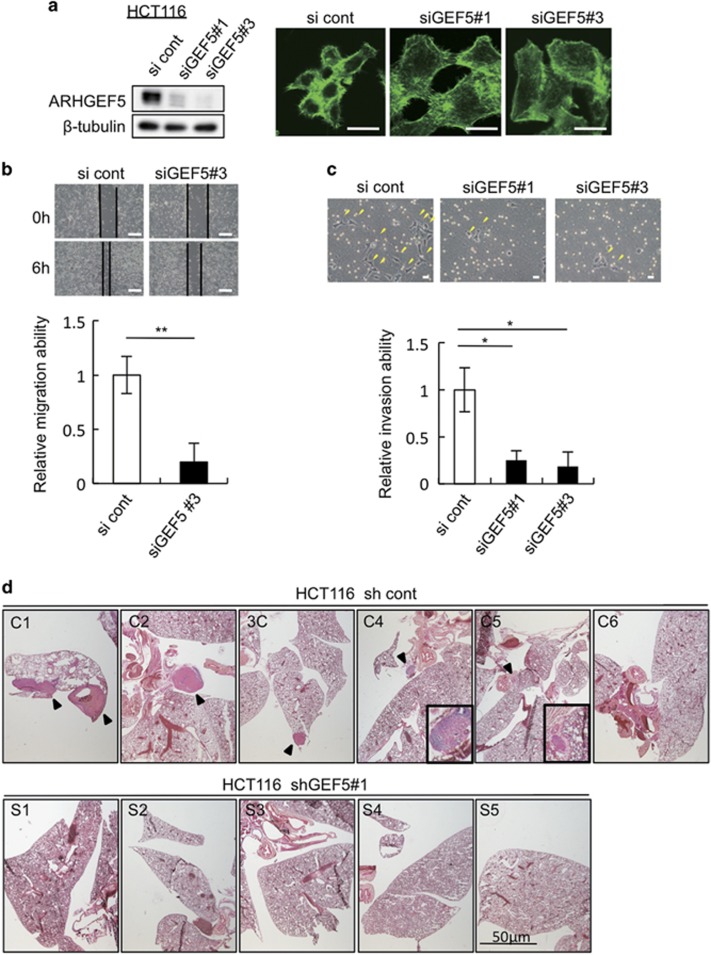
ARHGEF5 KD suppresses the invasive activity of human colorectal cancer HCT116 cells. (**a**) ARHGEF5 in HCT116 cells was knocked down with siRNAs (siGEF5 no. 1 and siGEF5 no. 3) in HCT116 cells and downregulation of ARHGEF5 confirmed by western blotting (upper). Control and siRNA-treated HCT116 were stained for F-actin. Scale bar: 20 μm. (**b**) Control and siRNA-treated HCT116 cells were subjected to an *in vitro* wound-healing assay. Scale bar: 200 μm. Values represent the mean±s.d. (*n*=3, **P<0.01). (**c**) The *in vitro* invasive activity of control HCT116 cells and those treated with specific siRNAs was examined in a Matrigel Transwell assay. Yellow arrowheads indicate invaded cells. Scale bar: 20 μm. Values represent the mean±s.d. (*n* = 3, *P<0.05). (**d**) The *in vivo* metastatic activity of HCT116 cells transfected with control and shGEF no. 1 was examined in experimental metastasis assays in nude mice. Metastatic lesions were observed by staining tissue slices with hematoxylin–eosin. Black arrowheads indicate metastatic lesions. Magnified views of the lesions in mice C4 and C5 are shown.

To evaluate the role of ARHGEF5 in human cancer cells, we examined the effects of ARHGEF5 KD on invasion and metastasis in human colorectal cancer HCT116 cells with invasive and metastatic activity. Immunofluorescence analysis showed that ARHGEF5 KD induced a rearrangement of the actin cytoskeleton (Figure 3a). Wound-healing and Transwell invasion assays revealed that ARHGEF5 KD significantly impaired cell migration (Figure 3b) and invasion (Figure 3c), respectively. Furthermore, in experimental metastasis assays in nude mice, control HCT116 cells formed metastatic lesions in the lungs of five out of six mice, whereas ARHGEF5 KD cells did not metastasize to the lungs in any of the mice examined (Figure 3d). These findings suggest that ARHGEF5 is involved in the invasive and metastatic activity of some human cancers.

### ARHGEF5 promotes tumor growth in mesenchymal-like cancer cells

**Figure 4:**
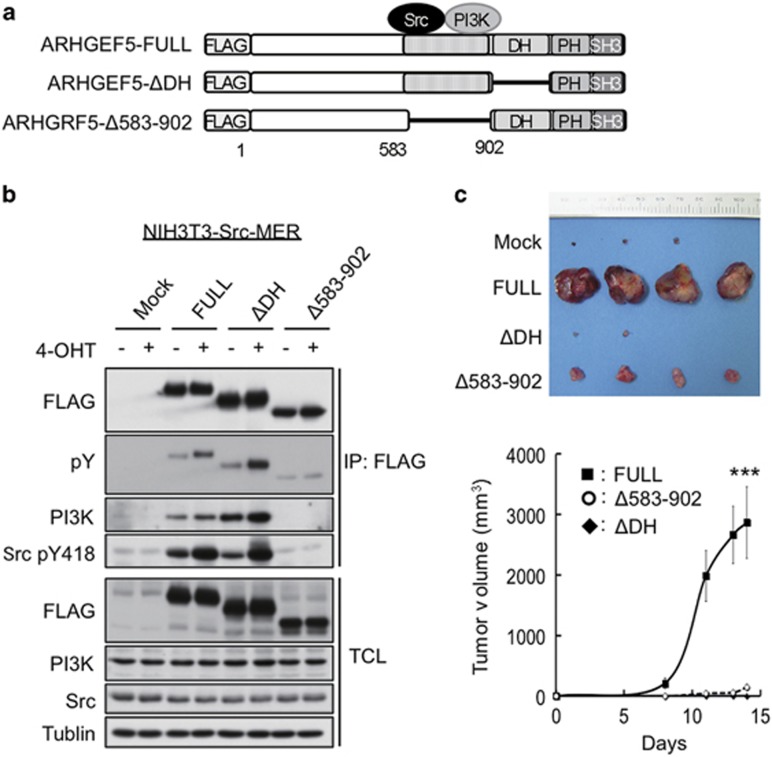
ARHGEF5 promotes tumorigenesis of Src-activated fibroblasts. (**a**) Schematic structures of wild-type ARHGEF5 (ARHGEF5-FULL) and its mutants lacking the DH domain (ΔDH) and Src/PI3K binding domain (Δ583–902). (**b**) NIH3T3 cells expressing the indicated constructs of ARHGEF5 were treated with or without 4-hydroxy tamoxifen (4-OHT) and ARHGEF5 was immunoprecipitated with an anti-FLAG antibody. Total cell lysates (TCLs) and the bound proteins were analyzed by western blotting. (**c**) NIH3T3 cells expressing the indicated ARHGEF5 constructs were subcutaneously inoculated into nude mice. Tumors generated 2 weeks after inoculation were excised and photographed (upper). Tumor length (*L*) and width (*W*) were measured and tumor volume was evaluated using the mathematical formula *V*=0.5 × *L* × *W*^2^ (lower). Values represent the mean±s.d. (*n*=4, ***P<0.001).

We previously showed that ARHGEF5 promotes anchorage-independent cell growth of Src-activated fibroblasts (NIH3T3-Src-MER cells).^21^ Xenograft assays in nude mice revealed that the expression of wild-type ARHGEF5 greatly promoted tumorigenesis in Src-activated fibroblasts, whereas mutant ARHGEF5 lacking GEF activity (ΔDH) or Src/PI3K (phosphatidylinositol 3-kinase) binding domain (Δ583–902) had no effect (Figures 4a–c). These observations suggest that ARHGEF5 has the potential to promote tumor growth via the Rho-ROCK and PI3K-Akt pathways.

**Figure 5:**
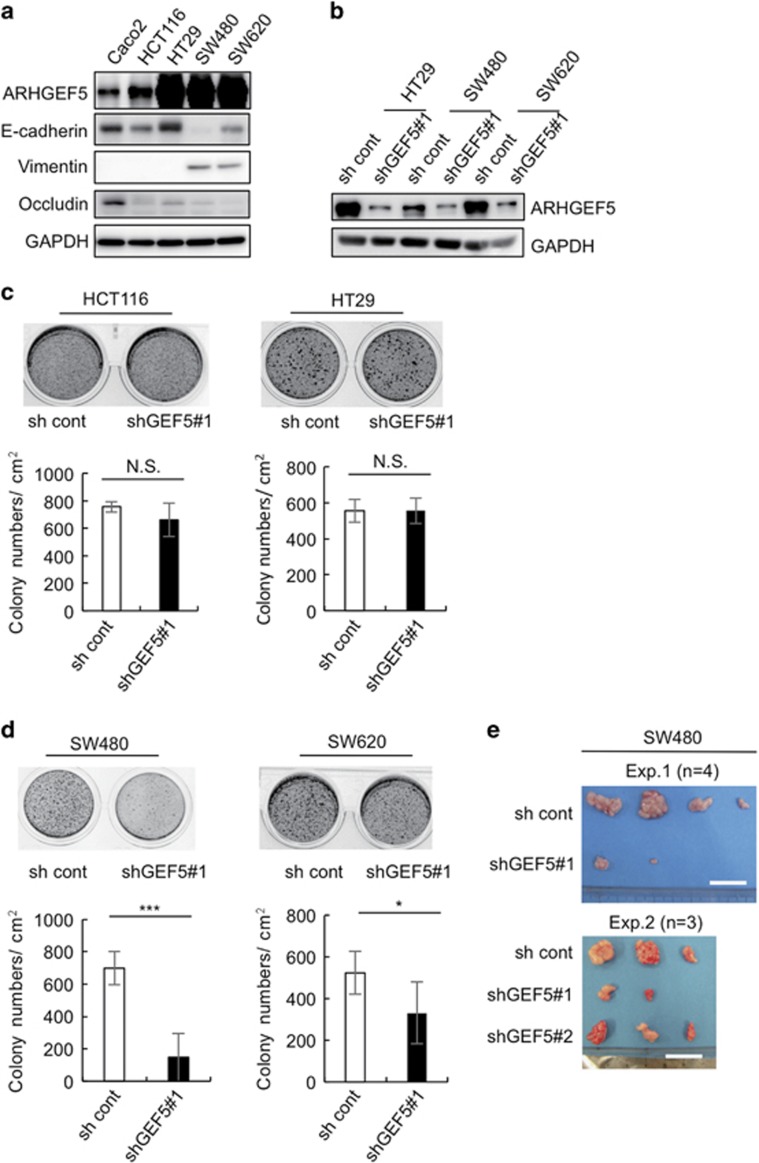
ARHGEF5 is required for tumor growth in mesenchymal-like cancer cells. (**a**) Expression of ARHGEF5 and the indicated EMT marker proteins in the indicated colorectal cancer cells was analyzed by western blotting. (**b**) ARHGEF5 in the indicated cells was stably knocked down by shRNA and the efficacy confirmed by western blotting. (**c**) Mock and ARHGEF5 KD HCT116 and HT29 cells were subjected to soft agar colony formation assays. Values represent the mean±s.d. (*n*=3; NS, not significant). (**d**) Mock and ARHGEF5 KD SW480 and SW620 cells were subjected to soft agar colony formation assays. Values represent the mean±s.d. (*n*=3, ***P<0.001, *P<0.05). (**e**) Mock and ARHGEF5 KD SW480 cells were subcutaneously inoculated into nude mice. Tumors generated 1 month after inoculation were excised and photographed. Tumors obtained from two independent experiments, Exp. 1 (*n*=4) and Exp. 2 (*n*=3), are shown. Scale bar: 2 cm.

To elucidate the role of ARHGEF5 in tumor growth from human colorectal cancer cells, we examined the effects of ARHGEF5 KD on anchorage-independent cell growth in epithelial-like HCT116 and HT29 cells as well as in mesenchymal-like SW480 and SW620 cells (Figures 5a and b). These cell types were categorized based on the expression of E-cadherin and vimentin (Figure 5a). The effects varied depending on cell type: ARHGEF5 KD did not affect the growth of epithelial-like HCT116 and HT29 cells (Figure 5c), but significantly suppressed the growth of mesenchymal-like SW480 and SW620 cells (Figure 5d). ARHGEF5 KD also suppressed *in vivo* tumorigenesis of SW480 cells (Figure 5e). These findings demonstrate that ARHGEF5 contributes to tumor growth, particularly in mesenchymal-like cancer cells, suggesting that the tumorigenic function of ARHGEF5 may be dependent on EMT status.

**Figure 6:**
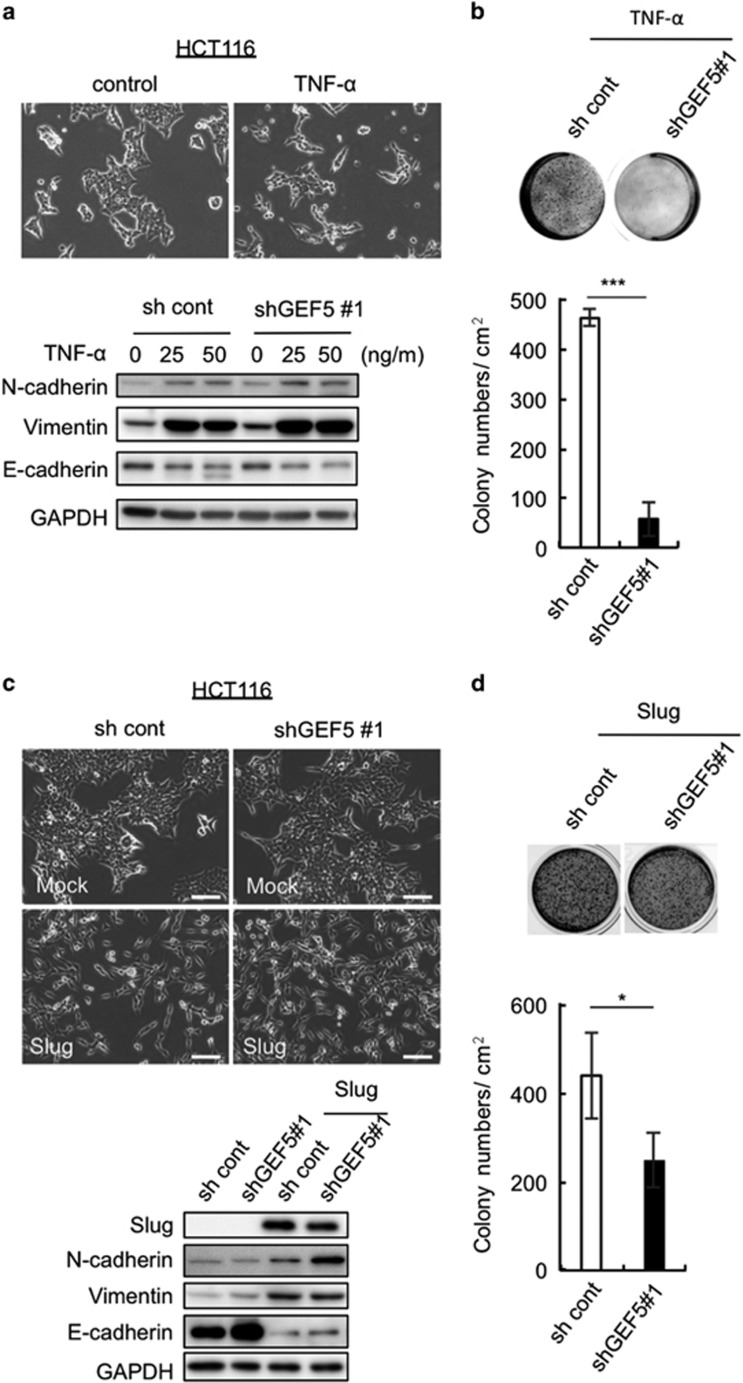
ARHGEF5 is required for tumor growth from HCT116 cells that have undergone EMT. (**a**) Mock and ARHGEF5 KD HCT116 cells were treated with TNF-α for 72 h, and expression of the indicated EMT markers was analyzed by western blotting (lower panels). (**b**) Mock and ARHGEF5 KD HCT116 cells treated with TNF-α were subjected to soft agar colony formation assays. Values represent the mean±s.d. (*n*=3, ***P<0.001). (**c**) Mock and ARHGEF5 KD HCT116 cells were stably transfected with or without myc-tagged Slug, and cell morphology observed (upper). Scale bar: 100 μm. The levels of the indicated EMT markers were analyzed by western blotting (lower). (**d**) HCT116 cells transfected with the indicated constructs were subjected to soft agar colony formation assays. Values represent the mean±s.d. (*n*=3, *P<0.05).

To explore this possibility, EMT was induced in epithelial-like HCT116 cells. Although TGF-β failed to induce EMT in these cells, TNF-α did, as determined by changes in cell morphology and the expression of N-canherin, vimentin and E-cadherin (Figure 6a). ARHGEF5 KD in TNF-α-treated cells potently suppressed anchorage-independent cell growth (Figure 6b). Furthermore, we forcedly induced EMT in these cells by overexpressing the pro-EMT transcription factor Slug^34^ (Figure 6c). Slug-induced EMT also sensitized these cells to growth suppression by ARHGEF5 KD (Figure 6d). These observations suggest that the tumorigenic functions of ARHGEF5 are activated when cancer cells acquire mesenchymal phenotypes via EMT.

### ARHGEF5-dependent activation of Akt is required for tumor growth in mesenchymal-like colorectal cancer cells

**Figure 7:**
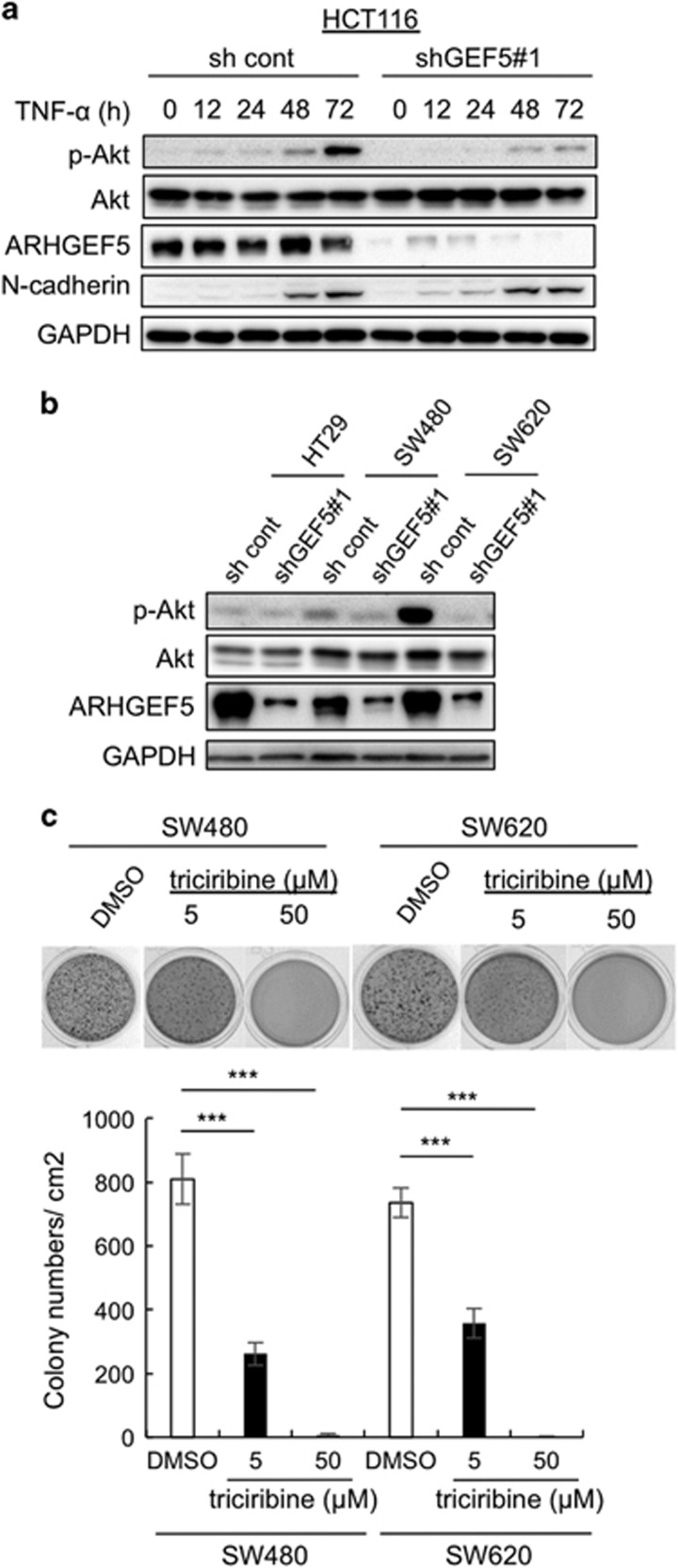
ARHGEF5-dependent activation of Akt is required for tumor growth from mesenchymal-like colorectal cancer cells. (**a**) HCT116 cells were treated with TNF-α (50 ng/ml) for the indicated periods, and the levels of the indicated proteins analyzed by western blotting. (**b**) HT29, SW480 and SW620 cells were transfected with control shRNA or shGEF5 no. 1 and the levels of the indicated proteins were analyzed by western blotting. (**c**) SW480 and SW620 cells treated with dimethyl sulfoxide (DMSO) or with the indicated concentrations of Akt inhibitor were subjected to soft agar colony formation assays. Values represent the mean±s.d. (*n*=3, ***P<0.001).

We then addressed the molecular mechanisms underlying the ARHGEF5-dependent tumor growth from mesenchymal-like cancer cells. Previously, we showed that ARHGEF5 interacts with PI3K,^21^ implicating ARHGEF5 in the regulation of the Akt pathway, which is tightly associated with tumor growth. We therefore investigated the impact of EMT status on the activity of Akt in HCT116 cells. During TNF-α-induced EMT, Akt was gradually activated in parallel with the induction of N-cadherin, and ARHGEF5 KD suppressed Akt activation (Figure 7a). Akt was in an active state in mesenchymal-like SW480 and SW620 cells, but not in epithelial-like HT29 cells, and ARHGEF5 KD attenuated Akt activation in mesenchymal-like cells (Figure 7b). Furthermore, the inhibition of Akt activity by the Akt inhibitor triciribine significantly suppressed the anchorage-independent growth of these cells (Figure 7c). These results suggest that Akt is activated via ARHGEF5 specifically in cells that have acquired mesenchymal phenotypes, thereby triggering cell signaling required for the promotion of tumor growth.

### ARHGEF5 upregulation associated with EMT-related gene expression is correlated with poor prognosis in patients with colorectal cancers

**Figure 8:**
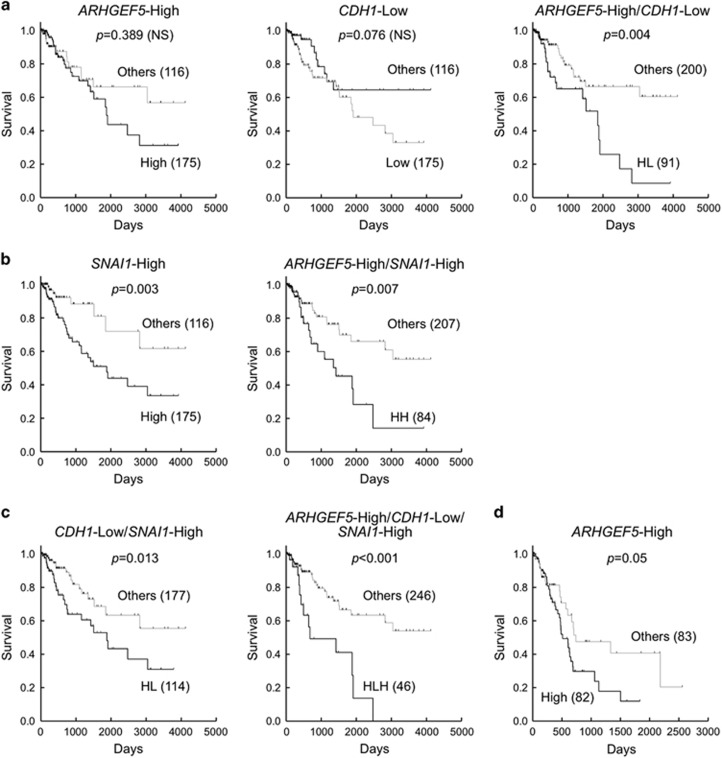
ARHGEF5 upregulation associated with EMT-related gene expression correlates with poor prognosis in patients with colorectal cancers. The correlation between ARHGEF5 and E-cadherin (CDH1) (**a**), ARHGEF5 and Snail (SNAI1) (**b**), and ARHGEF5, CDH1 and SNAI1 (**c**) expression and prognosis in colorectal cancer patients was estimated using the Kaplan–Meier method^42^ based on the transcriptome data set from the TCGA project. Statistical significance was calculated using the log-rank test. (**d**) The correlation between ARHGEF5 expression and the prognosis of pancreatic cancer patients was estimated.

To ascertain the relevance of ARHGEF5 in cancer patients, we examined the correlation between ARHGEF5 expression and prognosis in patients with colorectal cancers. To this end, we analyzed the transcriptome data set of colorectal cancer provided by The Cancer Genome Atlas (TCGA) project.^35^ Given the functional link between ARHGEF5 and EMT in our *in vitro* observations, we also investigated the expression of E-cadherin (CDH1) and Snail (SNAI1) to stratify the patients. In this study, patients with gene expression levels in upper 60% (ARHGEF5 and SNAI1) were defined as the 'High' group and those in lower 60% (CDH1) as the 'Low' group, whereas the remaining patients were designated as 'Others'. The ARHGEF5-High group showed a slight tendency towards a poorer prognosis than the Others, although the difference was not significant (Figure 8a). Similarly, the CDH1-Low group showed a tendency towards a poorer prognosis compared with the Others, although the difference was not statistically significant. However, in a combined analysis, the ARHGEF5-High/CDH1-Low (HL) group had a significantly worse prognosis than the Others. On the other hand, the SNAI1-High group by itself had a significantly poorer prognosis than the Others (Figure 8b). Combined analysis of ARHGEF5 and SNAI1 revealed that the prognosis of the ARHGEF5-High/SNAI1-High (HH) group was markedly worse than that of the Others. Furthermore, the ARHGEF5-High/CDH1-Low/SNAI1-High (HLH) group showed the best separation from the Others and the most significant statistical difference (Figure 8c). These data are consistent with the notion that EMT status correlate with the prognosis of colorectal cancers, and support our findings that increased activation of ARHGEF5 contributes to the progression of tumor malignancy in a manner dependent on EMT status.

## Discussion

Here we showed that ARHGEF5 has a pivotal role in malignant progression, namely the acquisition of invasive/metastatic properties and promotion of tumor growth, particularly in colorectal cancer cells that gained mesenchymal phenotypes via EMT. EMT is a crucial step in malignant progression as it involves the loss of cell polarity, detachment from the epithelial layer, migration and invasion. Dynamic cytoskeletal remodeling regulated by Rho GTPases is thought to be responsible for these processes;^36^ however, regulation of Rho GTPases activity during EMT remains unclear. We found that ARHGEF5 is upregulated during TGF-β-induced EMT and is required for activation of the RhoA-ROCK pathway. A previous study showed that ARHGEF5 expression is induced by Smad signals during TGF-β-induced mesenchymal transition in MS-1 endothelial cells (EndMT).^26^ Thus, expression of ARHGEF5 may be commonly regulated during EMT and EndMT through the TGF-β-Smad pathway. In the present study, Src and Fyn tyrosine kinases were upregulated during EMT in parallel with ARHGEF5 upregulation. Previous studies show that ARHGEF5 is phosphorylated by Src, causing a conformational change that leads to increased GEF activity toward RhoA.^21, 24^ Therefore, it is likely that Src/Fyn upregulation synergistically potentiates the activity of ARHGEF5, thereby promoting EMT via the Rho-ROCK pathway.^25^

The formation of podosomes/invadopodia has been implicated in the invasive and metastatic potential of cancer cells.^22, 23, 37^ Src activity is necessary for podosome formation, and active Rho, which localizes to podosomes, is required for the assembly of these structures.^38^ We identified ARHGEF5 as a GEF responsible for the activation of podosomal Rho,^21^ and an extended analysis using ARHGEF5 knockout mouse embryonic fibroblasts corroborated that ARHGEF5 accumulates in podosomes and is essential for Src-induced podosome formation (Supplementary Figure S4). Thus, the EMT-mediated upregulation of the Src-ARHGEF5-Rho axis may contribute to the acquisition of invasive and metastatic properties by promoting podosome/invadopodia formation.

The present study showed that ARHGEF5 is crucial for tumor growth, particularly in mesenchymal-like cells. ARHGEF5 KD inhibited tumor growth from mesenchymal-like colorectal cancer SW480 and SW620 cells, whereas it failed to suppress the growth of epithelial-like HCT116 and HT29 cells. These observations suggest that the acquisition of mesenchymal phenotypes is required for the tumorigenic function of ARHGEF5. In support of this notion, forcedly induction of EMT in HCT116 cells sensitized these cells to growth suppression by ARHGEF5 KD. Mesenchymal cells generated by EMT form cell adhesion sites, that is, focal adhesions and/or podosomes/invadopodia, at which the Src-ARHGEF5-Rho axis is upregulated and activated.^21^ As ARHGEF5 can also function as a scaffold for PI3K,^21^ upregulated ARHGEF5 may activate Akt pathways, thereby promoting cell survival via the antiapoptotic pathway and cell growth via the mammalian target of rapamycin complex 1 pathway.^39^ Indeed, we observed that ARHGEF5-dependent activation of Akt was required for tumor growth from mesenchymal-like colorectal cancer cells. These results suggest that the EMT-mediated assembly of the ARHGEF5 axis at cell adhesion sites have a crucial role in promoting both cell invasion/metastasis and tumor growth in mesenchymal-like cancer cells (Supplementary Figure S5).

To extend the EMT-dependent function of ARHGEF5 to human cancer patients, we investigated the correlation between ARHGEF5/EMT markers and prognosis in patients with colorectal cancers. Although ARHGEF5 expression alone did not correlate significantly with poor prognosis, patients with high ARHGEF5 expression in the CDH1-Low group had a remarkably poorer prognosis than the other patients. Similarly, the ARHGEF5-High/SNAI1-High group had a much poorer prognosis than the other patients. Notably, the ARHGEF5-High/CDH1-Low/SNAI1-High group had the worst prognosis in all settings. These findings support the idea that the functions of ARHGEF5 depend on EMT status, even in human colorectal cancers. In pancreatic cancers, however, the single ARHGEF5-High group had a significantly poorer prognosis comapred with the Others (Figure 8d), supporting the important role of ARHGEF5 in this tumor type. On the other hand, there was no significant correlation between ARHGEF5/EMT and prognosis in breast cancers that typically invade as an epithelial multicellular unit^40^ (data not shown). These findings suggest that, although the contribution of ARHGEF5/EMT varies depending on tumor types and their strategies for invasion and metastasis, ARHGEF5 and related molecules may represent potential targets for the treatment of a subset of malignant tumors that have undergone EMT.

## Materials and methods

### Reagents and antibodies

Anti-ARHGEF5 was generated in rabbits via immunization with GST-mouse or human ARHGEF5 (amino acids 2–204) and affinity purified using a maltose-binding protein-tagged antigen. Anti-Src-pY418, anti-GFP, anti-FAK-pY397, SD208, Alexa Fluor 488 phalloidin, Alexa Fluor 594-conjugated goat anti-rabbit immunoglobulin G, horse radish peroxidase-conjugated goat anti-rabbit immunoglobulin G, anti-mouse immunoglobulin G, anti-occludin and anti-cortactin-pY421 were purchased from Thermo Fisher Scientific (Waltham, MA, USA). Anti-GAPDH, anti-Fyn, anti-Lyn and anti-vimentin were from Santa Cruz Biotechnology (Santa Cruz, CA, USA). Anti-v-Src, anti-cortactin (4F11) and anti-phosphotyrosine (4G10) were from Millipore (Billerica, MA, USA). Anti-FLAG (M2) and anti-β-tubulin were from Sigma-Aldrich (St Louis, MO, USA). Anti-E-cadherin, anti-N-cadherin and anti-FAK were from BD Transduction Laboratories (Lexington, KY, USA). Anti-Smad2-pS465/467, anti-Smad2, anti-MLC2-pT18/S19, anti-MLC2, anti-Akt and anti-Akt-pS473 were from Cell Signaling Technology Inc. (Beverly, MA, USA). TGF-β1 and TNF-α were from PeproTech (Rocky Hill, NJ, USA). The Akt inhibitor triciribine was from Selleckchem (Houston, TX, USA).

### Cell culture

The normal breast epithelial cell line MCF10A and the human colon cancer cell lines Caco-2, SW480, SW620, HCT116 and HT29 were purchased from the American Type Culture Collection (Manassas, VA, USA). ARHGEF5 knockout cells were established by transfecting ARHGEF5^fl/fl^ mouse embryonic fibroblasts with a Cre vector (gifted by Dr Masahito Ikawa, Osaka University, Suita, Japan). All cells were cultured at 37 °C in a humidified atmosphere containing 5% CO_2_. MCF10A cells were maintained in Dulbecco's modified Eagle's medium (DMEM) supplemented with 5% horse serum, Ham's F12 Nutrient Mixture (Thermo Fisher Scientific), 20 ng/ml epidermal growth factor, 100 ng/ml cholera toxin, 500 ng/ml hydrocortisone and 5 μg/ml insulin. SW480, SW620, HCT116 and HT29 cells were maintained in DMEM supplemented with 10% (v/v) fetal bovine serum and penicillin/streptomycin. NIH3T3 cells harboring the Src-MER system were generated as described previously,^21^ and maintained in DMEM supplemented with 10% (v/v) fetal bovine serum.

### Plasmid and siRNA constructs

ARHGEF5 cDNA with a 3xFLAG tag was subcloned into the pCX4-bsr vector (gifted by Dr Tsuyoshi Akagi, KAN Research Institute, Kobe, Japan). ARHGEF5 GFP, ARHGEF5 ΔDH (deleted amino acids, 1158–1341) and ARHGEF5 were subcloned into the pCX4-bsr vector. Myc-tagged Snail and Slug cDNAs were subcloned into the pCX4-bleo vector. Lentiviral vectors carrying sh-ARHGEF5 no. 1 (shGEF5 no. 1) and sh-ARHGEF5 no. 2 (shGEF5 no. 2) were purchased from Sigma-Aldrich. The series of ARHGEF5 siRNA duplexes (siGEF5 no. 1–3) (Stealth, MSS225557) and Stealth siRNA Negative Control (12935-112) were purchased from Invitrogen (Carlsbad, CA, USA). The sequences of short hairpin RNA (shRNAs) and siRNAs are as follows: shGEF5 no. 1, 5′-CCGGGCAACATGACAAACTTCCTATCTCGAGATAGGAAGTTTGTCATGTTGCTTTTT-3′ shGEF5 no. 2, 5′-CCGGCTCTCAAGAATCCATCTCAAACTCGAGTTTGAGATGGATTCTTGAGAGTTTTT-3′ siGEF5 no. 1, 5′-UUCAGAGGAAGGAUCUAUGAUAGGGCCCUAUCAUAGAUCCUUCCUCUGAA-3′ siGEF5 no. 2, 5′-UAAGCAGUUCACUUCCACUGCCCUGCAGGGCAGUGGAAGUGAACUGCUUA-3′ siGEF5 no. 3, 5′-UGUAUUAUUAAAUUCCUCCUGAGGGCCCUCAGGAGGAAUUUAAUAAUACA-3′.

### RT–PCR and primers

Total RNA was prepared using Sepazol Super G (Nacalai Tesque, Kyoto, Japan). Reverse transcription was carried out using the Transcriptor First Strand cDNA Synthesis Kit (Roche, Basel, Switzerland). PCR was performed using the following primers: ARHGEF5 forward, 5′-CAGTCCTGCTGAAGCCTACC-3′ ARHGF5 reverse, 5′-GGGAACCACTACACGAGCAT-3′ ARHGF5 reverse, 5′-GGGAACCACTACACGAGCAT-3′ GAPDH forward, 5′-CGAGATCCCTCCAAAATCAA-3′ and GAPDH reverse, 5′-TGCTGTAGCCAAATTCGTTG-3′.

### Retroviral and lentiviral gene transfer, and lipofection

Gene transfer experiments were performed using the pCX4 series of retroviral vectors.^41^ pCX4 vectors were transfected into PLT cells using FuGENE (Roche) and the culture supernatant used as the source of virus. KD of ARHGEF5 was performed using lentiviral vectors. Lentiviruses were generated from PLT cells using the MISSION Lentiviral packaging mix (Sigma-Aldrich) and FuGENE. siRNA and cDNA were transiently transfected using RNAimax and Lipofectamine 3000 (Thermo Fisher Scientific), respectively. The active form of Src (SrcF572) was introduced into mouse embryonic fibroblasts and MCF10A cells using the Retro-X Tet-On 3G Inducible Expression System (Clontech, Mountain view, CA, USA).

### Western blotting

Cells were lysed in sodium dodecyl sulfate sample buffer (2% sodium dodecyl sulfate, 62.5 mm Tris-HCl (pH 6.8), 5% sucrose). Equal amounts of total proteins were separated by sodium dodecyl sulfate–polyacrylamide gel electrophoresis and transferred onto polyvinylidene difluoride membranes. Membranes were blocked and incubated with primary antibodies, followed by incubation with horse radish peroxidase-conjugated secondary antibodies. Signals were visualized on a WSE6200H luminograph II (ATTO, Tokyo, Japan). Representative blots were obtained from at least three independent experiments are shown.

### Soft agar colony formation assay

DMEM (1.25 ml) supplemented with 10% fetal bovine serum and 0.7% Bacto-Agar (BD Transduction Laboratories) was placed in each well of a 12-well plate (bottom layer). After the agar solidified, cells (in 1 ml of culture medium+0.36% agar) were poured onto the bottom layer. After 5–14 days, colonies were fixed and stained with 1 mg/ml MTT (3-(4,5-dimethylthiazol-2-yl)-2,5-diphenyltetrazolium bromide). Colonies were counted using ImageJ (National Institute of Health, Bethesda, MD, USA).

### Immunohistochemistry

Cells were seeded on a 12 mm coverslips coated with 5 μg/ml fibronectin. The samples were fixed in 4% paraformaldehyde, and permeabilized with 0.1% Triton-X in PBS (T-PBS). Samples were blocked with 1% bovine serum albumin in T-PBS, and then incubated with primary antibody overnight at 4 °C, followed by incubation with secondary antibody at room temperature. After the samples were washed with T-PBS, coverslips were mounted on glass slides using ProLong Gold (Thermo Fisher Scientific). The next day, the coverslips were sealed with ProLong Gold Antifade reagent and observed under a confocal microscope (FV1000; Olympus, Tokyo, Japan). The same experiments were repeated at least three times.

### Invasion assay

BioCoat Matrigel Invasion Chambers (BD Biosciences, Franklin Lakes, NJ, USA) were used for the invasion assay. Cells (1 × 10^5^ for LuM1 and NM11, or 5 × 10^4^ for HCT116) were seeded on inserts and moved into chambers containing culture supernatant of NIH3T3 cells. After incubation at 37 °C for 24 h (LuM1 or NM11) or 48 h (HCT116), invaded cells were fixed with 100% methanol and then stained with 0.1% toluidine blue. Invaded cells were counted on micrographs; in each experiment, cells were counted on five randomly chosen fields. Experiments were repeated at least three times.

### *In vivo* metastasis assay

HCT116 cells transfected with or without ARHGEF5 shRNA (3 × 10^6^ in 100 μl of serum-free medium) were intravenously injected into six nude mice (BALB/c Slc-nu/nu, 4 weeks old, female). After 7 weeks, the surviving mice were killed and their lungs removed. Metastatic lesions were detected by staining the tissue slices with hematoxylin–eosin in a blind test. Mice were handled and maintained according to the Osaka University guidelines for animal experimentation.

### *In vivo* tumorigenicity assay

NIH3T3-Src-MER cells treated with 4-hydroxy tamoxifen and SW480 cells transfected with or without ARHGEF5 shRNAs (4 × 10^6^ in 200 μl of serum-free medium) were subcutaneously injected into nude mice (BALB/c Slc-nu/nu, 4 weeks old, female). After 2–4 weeks, the surviving mice were killed, their tumors were removed and the sizes and weight of the tumors were measured. Mice were handled and maintained according to the Osaka University guidelines for animal experimentation.

### Kaplan–Meier survival analysis

Clinical and RNA-seq data from the publically available TCGA data set from 291 colon adenocarcinoma or 165 pancreatic adenocarcinoma patients were used for analysis of ARHGEF5, E-cadherin and Snail expression in tumor samples. Survival curves were estimated using the Kaplan–Meier method, and the obtained survival curves were compared by using the log-rank test.
