# Supplementary material for: The Rho guanine nucleotide exchange factor ARHGEF5 promotes tumor malignancy via epithelial–mesenchymal transition
Source: Oncogenesis. 2016 Sep 12;5(9):e258–. doi: 10.1038/oncsis.2016.59 (PMC5047960; doi:10.1038/oncsis.2016.59)
Supplement: Supplementary Information [file oncsis201659x1.pdf]

Supplementary information

Supplementary figure legends

Figure S1. EMT is induced by TGF-β in MCF10A cells. (A) MCF10A cells were treated with or without TGF-β1 for 48 h and stained for E-cadherin, N-cadherin, and F-actin. Cell morphology was observed under a phase-contrast microscope (PC). White arrowheads in the F-actin images indicate the locations of podosome-like structures. Scale bar: 20 μm. (B) MCF10A cells were treated with TGF-β1 for the indicated periods, and the levels of the indicated proteins analyzed by western blotting. (C) MCF10A cells treated with or without TGF-β1 were subjected to wound-healing assays. Scale bar: 100 μm. Values represent the mean ± SD (n=3, ***p*<0.01).

Figure S2. The ROCK inhibitor, Y27632, suppresses TGF-β-induced EMT phenotypes in MCF10A cells. (A) MCF10A cells were treated with TGF-β for 48 h in the presence or absence of Y27632 and the cells stained with anti-E-cadherin or anti-N-cadherin. Scale bar: 20 μm. (B) MCF10A cells were treated with TGF-β for 48 h in the presence or absence of Y27632 and then subjected to wound-healing assays. Scale bar: 200 μm. (C) The migration rates of the indicated cells are shown. Values represent the mean ± SD (n=3, ****p*<0.001). (D) MCF10A cells overexpressing Flag-ARHGEF5 were treated with Y27632 for 24h and MLC phosphorylation and the indicated proteins was analyzed by western blotting.

Figure S3. ARHGEF5 KD does not affect expression of EMT-related transcription factors. Mock and ARHGEF5-KD MCF10A cells were treated with or without TGF-β for the indicated periods and expression of the indicated EMT-related transcription factors was assessed by RT-PCR. PCR was performed with the following primers:
ACTA2 forward, 5'-TTCAATGTCCCAGCCATGTA-3';
ACTA2 reverse, 5'-GAAGGAATAGCCACGCTCAG-3';
SNAI1 forward, 5'-TTTACCTTCCAGCAGCCCTA-3';
SNAI1 reverse, 5'-CCCACTGTCCTCATCTGACA-3';
SNAI2 forward, 5'-TCGGACCCACACATTACCTT-3';
SNAI2 reverse, 5'-TTGGAGCAGTTTTTGCACTG-3';
SNAI3 forward, 5'-ACTGCCACAAACCCTACCAC-3';
SNAI3 reverse, 5'-ATAGGGCTTCTCCCCTGTGT-3';
TWIST1 forward, 5'-GTCCGCAGTCTTACGAGGAG-3';
TWIST1 reverse, 5'-TGGAGGACCTGGTAGAGGAA-3';
TWIST2 forward, 5'-AGCAAGAAGTCGAGCGAAGA-3';
TWIST2 reverse, 5'-CAGCTTGAGCGTCTGGATCT-3';
ZEB1 forward, 5'-TGCACTGAGTGTGGAAAAGC-3';

ZEB1 reverse, 5'-TGGTGATGCTGAAAGAGACG-3';
ZEB2 forward, 5'-CGCTTGACATCACTGAAGGA-3'; and
ZEB2 reverse, 5'-CTTGCCACACTCTGTGCATT-3'.

Figure S4. Arhgef5 is required for Src-induced podosome formation. The role of Arhgef5 in Src-induced transformation was confirmed using Arhgef5-knockout mouse embryonic fibroblasts (KO MEFs) harboring a Tet-on inducible system of active Src. In wild-type MEFs (WT), Src upregulation induced the formation of podosome rings in which phospho-cortactin and F-actin were enriched. Arhgef5 KO markedly suppressed podosome ring formation, while re-expression of Arhgef5, but not ΔDH lacking the GEF domain, restored podosome ring formation. Furthermore, localization analysis using Arhgef5-GFP revealed that Arhgef5 accumulates in podosomes. These results corroborated the essential role of Arhgef5 in Src-induced podosome formation in fibroblasts. (A) WT or Arhgef5 KO MEFs expressing mock vector, Arhgef5, and ΔDH, all harboring a Tet-on system for active Src, were treated with Dox for the indicated periods. Cells were then stained for phospho-cortactin and F-actin. The insets in the bottom panels are magnified views of podosome rings. Scale bar: 20 μm. (B) The average number of podosome rings formed per cell for the indicated cell lines is indicated. Values represent the mean ± SD (n=50, ***p*<0.01). (C) Arhgef5 KO MEFs expressing the Arhgef5-GFP construct were stained for F-actin (upper) and GFP signals observed (lower). White arrowheads indicate the locations of podosome rings. Scale bar: 10 μm.

Figure S5. Model of ARHGEF5 functions. EMT-meditated upregulation of the Src-ARHGEF5-Rho axis results in formation of a signaling platform at focal adhesions and/or podosome/invadpodia, which triggers cell signaling and promotes invasion/metastasis and tumor growth.

Suppl. Fig. S1

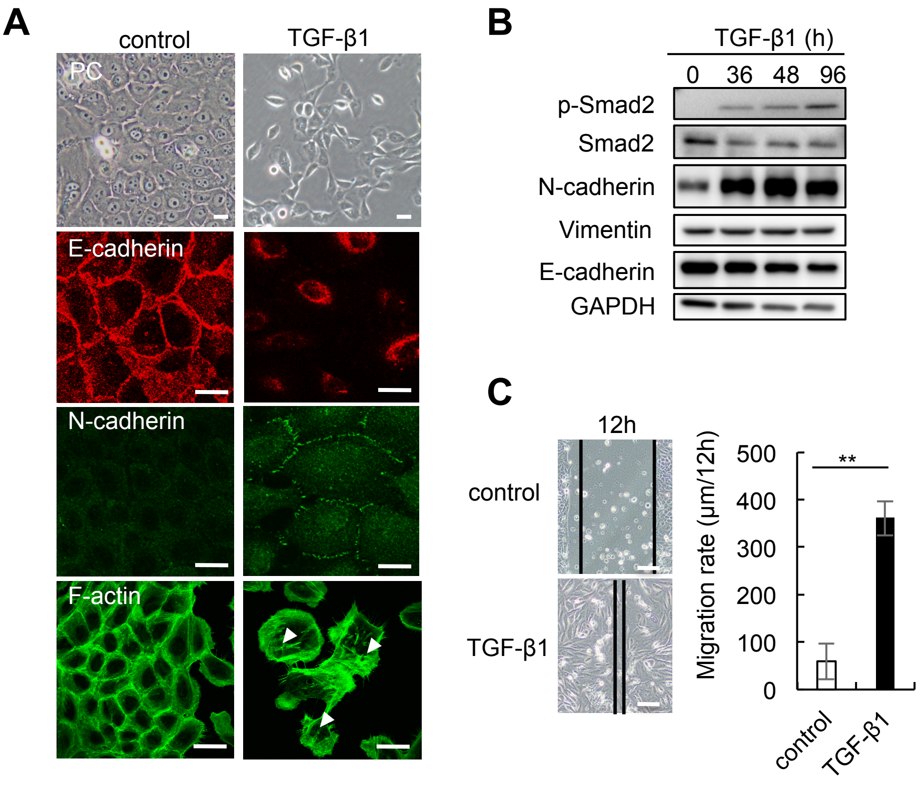

A
control
TGF-β1
PC
E-cadherin
N-cadherin
F-actin
B
TGF-β1 (h)
0
36
48
96
p-Smad2
Smad2
N-cadherin
Vimentin
E-cadherin
GAPDH
C
12h
control
TGF-β1
Migration rate (μm/12h)
500
400
300
200
100
0
**
control
TGF-β1

Suppl. Fig. S2

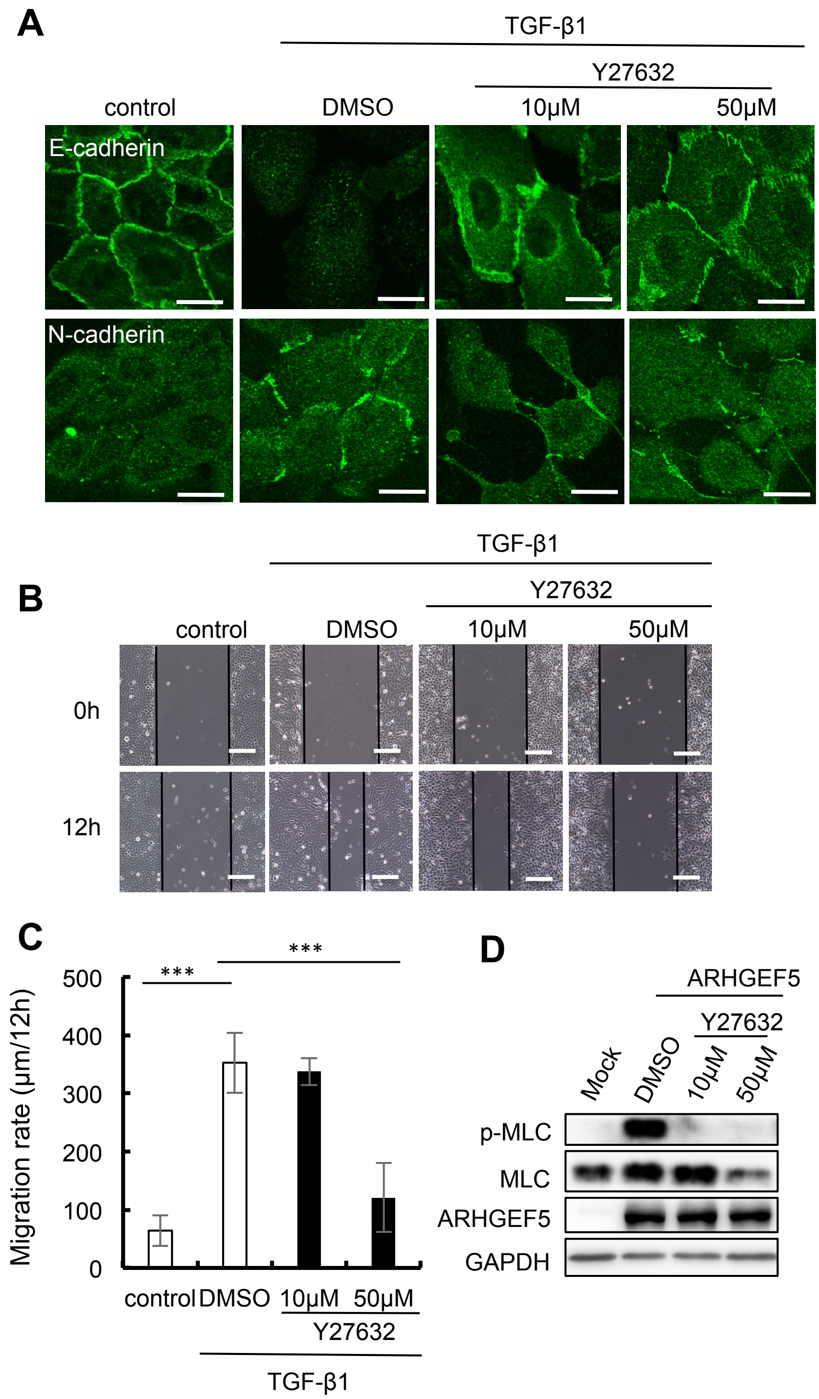

A
TGF-β1
Y27632
control
DMSO
10μM
50μM
E-cadherin
N-cadherin
B
TGF-β1
Y27632
control
DMSO
10μM
50μM
0h
12h
C
***
***
Migration rate (μm/12h)
500
400
300
200
100
0
control
DMSO
10μM
50μM
Y27632
TGF-β1
D
ARHGEF5
Y27632
Mock
DMSO
10μM
50μM
p-MLC
MLC
ARHGEF5
GAPDH

Suppl. Fig. S3

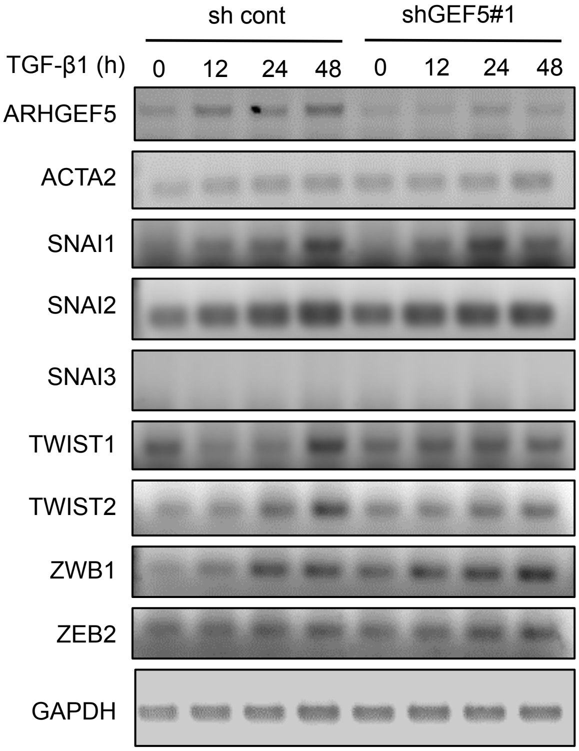
sh cont
shGEF5#1
TGF-β1 (h)
0
12
24
48
0
12
24
48
ARHGEF5
ACTA2
SNAI1
SNAI2
SNAI3
TWIST1
TWIST2
ZWB1
ZEB2
GAPDH

Suppl. Fig. S4

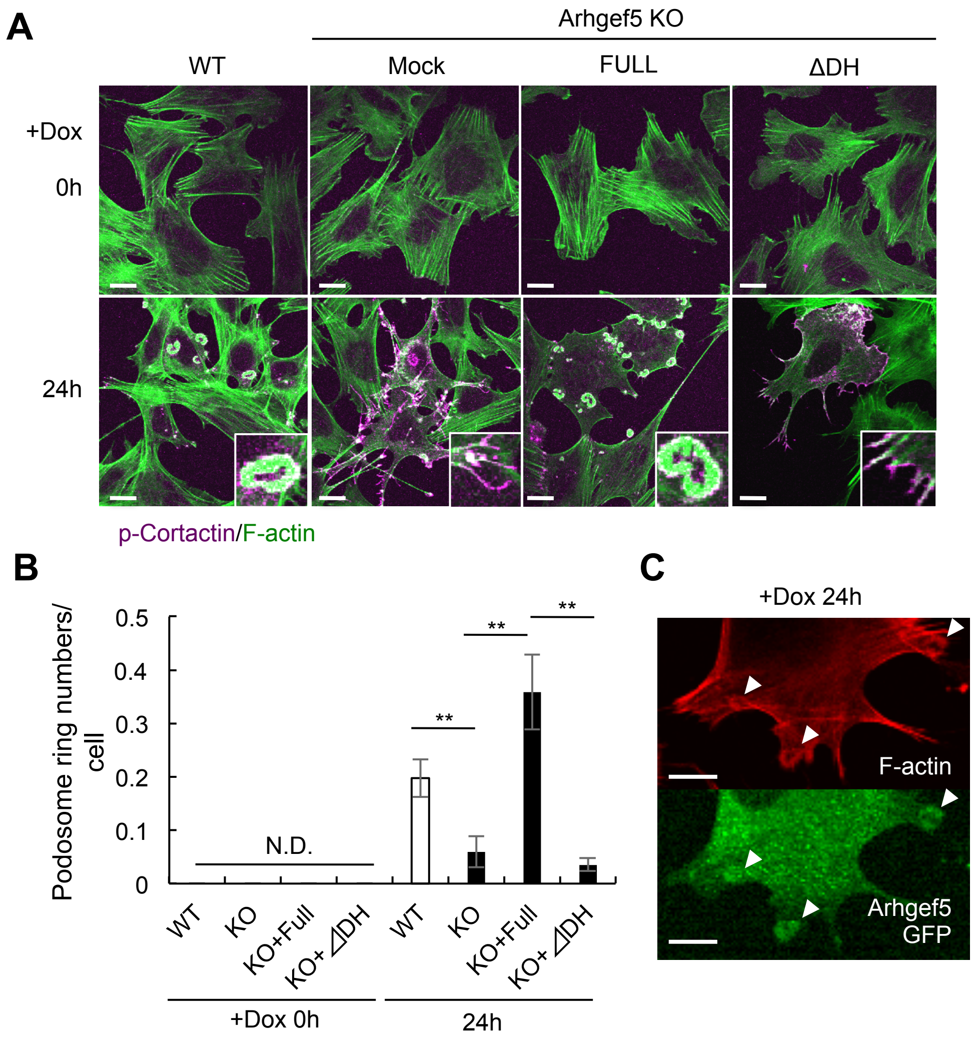
A
Arhgef5 KO
WT
Mock
FULL
ΔDH
+Dox
0h
24h
p-Cortactin/F-actin
B
Podosome ring numbers/cell
0.5
0.4
0.3
0.2
0.1
0
N.D.
**
**
**
WT
KO
KO+Full
KO+⊿DH
WT
KO
KO+Full
KO+⊿DH
+Dox 0h
24h
C
+Dox 24h
F-actin
Arhgef5
GFP

Fig. S5

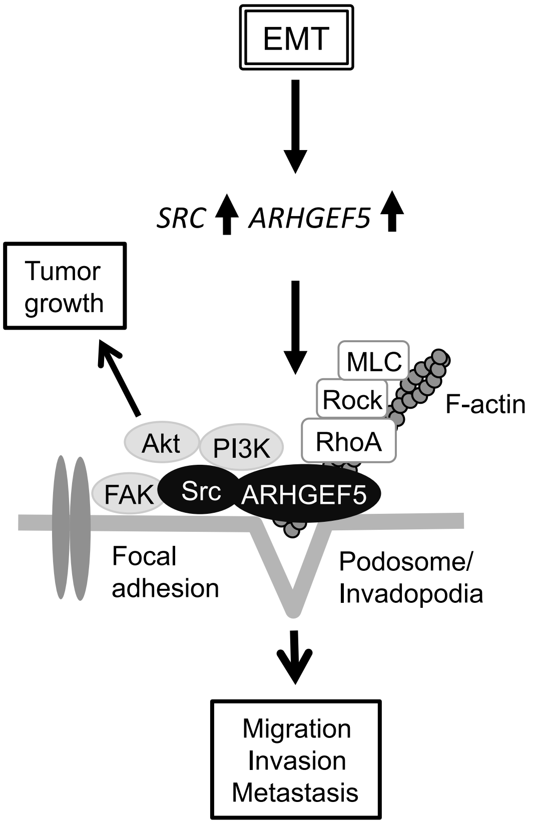

EMT
SRC
ARHGEF5
Tumor growth
MLC
Rock
F-actin
RhoA
Akt
PI3K
FAK
Src
ARHGEF5
Focal adhesion
Podosome/ Invadopodia
Migration
Invasion
Metastasis
